# Targeting Nrf2 may reverse the drug resistance in ovarian cancer

**DOI:** 10.1186/s12935-021-01822-1

**Published:** 2021-02-17

**Authors:** Danjie Li, Xiaoling Hong, Feijie Zhao, Xinxin Ci, Songling Zhang

**Affiliations:** 1 Department of Obstetrics and Gynecology, The First Hospital of Jilin University, Changchun, China; 2 Institute of Translational Medicine, The First Hospital of Jilin University, Changchun, China

**Keywords:** *Nrf2*, Drug resistance, Reactive oxidative stress, Ovarian cancer

## Abstract

**Background:**

Acquired resistance to therapeutic drugs has become an important issue in treating ovarian cancer. Studies have shown that the prevalent chemotherapy resistance (cisplatin, paclitaxel etc.) for ovarian cancer occurs partly because of decreased production of reactive oxygen species within the mitochondria of ovarian cancer cells.

**Main Body:**

Nuclear erythroid-related factor-2 (*Nrf2*) mainly controls the regulation of transcription of genes through the *Keap1-Nrf2-ARE* signaling pathway and protects cells by fighting oxidative stress and defending against harmful substances. This protective effect is reflected in the promotion of tumor cell growth and their resistance to chemotherapy drugs. Therefore, inhibition of the *Nrf2* pathway may reverse drug resistance. In this review, we describe the functions of *Nrf2* in drug resistance based on *Nrf2*-associated signaling pathways determined in previous studies.

**Conclusions:**

Further studies on the relevant mechanisms of *Nrf2* may help improve the outcomes of ovarian cancer therapy.

## Background

Malignant ovarian tumors are one of the most common malignant tumors of the female reproductive organ. Among them, ovarian epithelial cancer has the highest mortality rate, posing a serious threat to women’s life. Early stage ovarian tumors are usually located deep inside the pelvis, exhibit no typical symptoms and are thus discovered only at the advanced stage. The treatment options for advanced ovarian cancer are usually cytoreductive surgery and chemotherapy. However, the current chemoresistance in ovarian cancer(OC)has become a key cause of treatment failure and OC-related deaths [1]. Although extensive research has been carried out on complex factors, including increased drug efflux, drug inactivation, alteration in drug target, and increased DNA repair, the existing mechanisms fail to completely account for the drug resistance in OC [2, 3]. In recent years, the level of oxygen species (ROS) has also been reported to play a vital role in the development of drug resistance in OC, and thus targeting ROS levels may be a promising strategy to conquer cancer chemoresistance.

Oxidative stress refers to the process of oxidative damage caused by an imbalance between the production and scavenging of oxygen free radicals in the body or cells, resulting in the accumulation of ROS and RNS in the body or cells. Increased ROS levels activate relevant signaling pathways, inhibit the function of tumor suppressor genes, and induce oncogenic mutations, ultimately leading to tumorigenesis [4, 5]. Moreover, the significance of elevated ROS lies in facilitating genomic instability and DNA damage in tumors with drug resistance and recurrence [6, 7]. Consequently, more researches on ROS regulation would assist us to overcome drug resistance in OC.

*Nrf2* exerts a modifying influence on cellular oxidative stress response. At the same time, by modulating the expression of antioxidant genes, *Nrf2* can help prevent cell damage from ROS and electrophiles and keep the balance of intracellular redox homeostasis [8, 9]. Conversely, findings from previous studies suggest that continuous activation of antioxidant *Nrf2* may be beneficial to the growth of cancer cells, and may become a way for cancer cells to escape the attack of chemotherapy drugs, providing conditions for cancer cells to develop drug resistance [7, 10–12]. Accordingly, the purpose of this review is to review recent research on *Nrf2*-related drug resistance and mechanisms in OC to provide reference for clinical treatment.

## *Nrf2 *and ROS

### ROS regulation in OC

Recently, several studies showed that the generation of ROS is associated with drug resistance [13–16]. On the one hand that ROS mediate cytotoxicity induced by drugs in tumor cells. On the other hand ,cancer cells are surrounded by antioxidant molecules to keep ROS in the tumor microenvironment (TME), which contributes to the maintenance of drug resistance in OC [17]. This phenomenon may be caused by the different concentrations of ROS in cancer cells [18]. Usually, at low levels, ROS stimulate cell proliferation and survival in the form of mitogens [19, 20]. At medium levels, ROS may hinder the cell cycle process at varying degrees and induce cell differentiation [21]. At high levels, ROS might impair fundamental cellular substances such as proteins, DNAs, RNAs, and cause gene mutations—inhibition of tumor suppressor genes(*P53,PTEN*)and activation of oncogenes(*K-ras, ERK,AKT*), resulting in tumorigenesis in normal cells or multidrug resistance in cancers [18]. Consistently, Meng et al. and Dharmaraja et al. have identified that platinum-resistant OC cells can maintain steady high levels of ROS,which results in DNA damage [13, 22, 23]. In addition, several studies have indicated that in the TME, hypoxia-induced ROS cause cisplatin resistance by downregulating* p-Drp1 (Ser637)* and* Mfn1 *in OC cells [15, 16]. Common radio- and chemotherapeutic agents affect tumor outcome by modulating ROS; therefore, the impact of ROS modulation is essential for cancer treatment.

## *Nrf2* regulation in OC

**Fig. 1:**
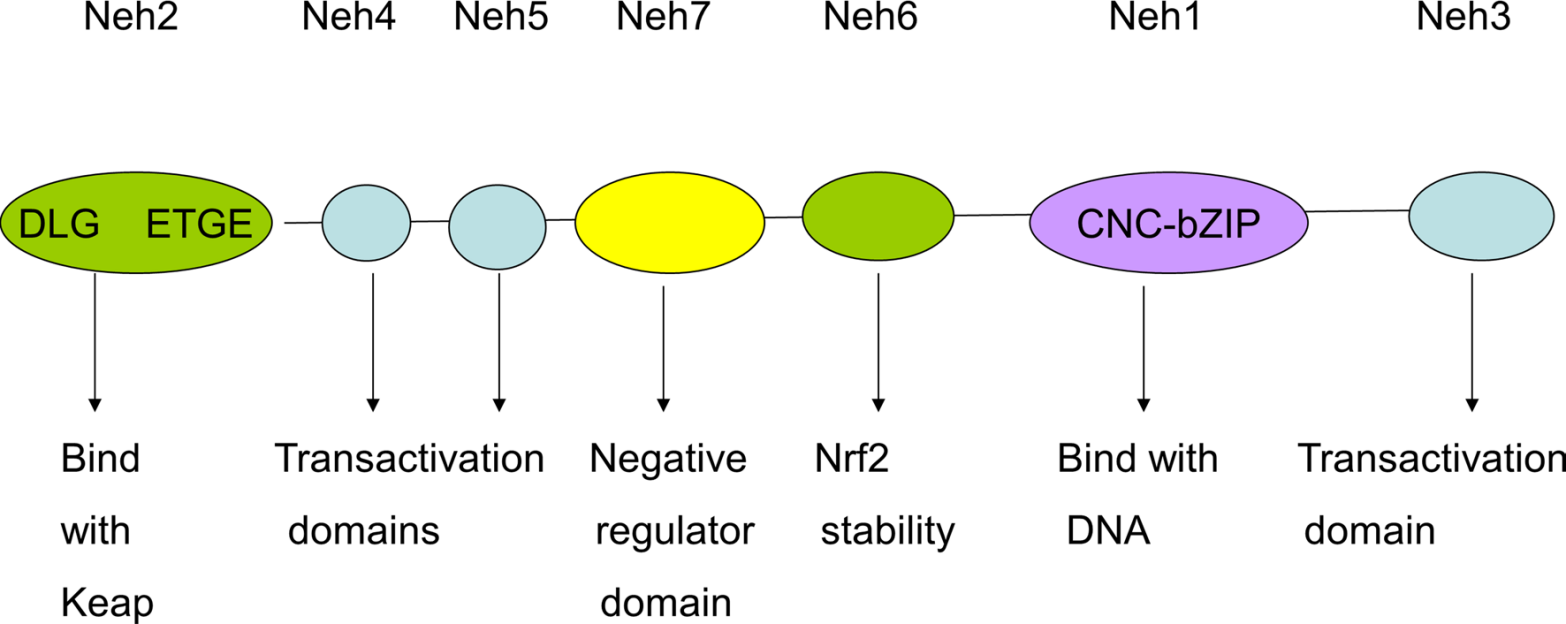
Structure of the human *Nrf2* protein. The Neh1 domain consists of the *CNC-bZIP* region, in which DNA binds the sMaf proteins as *Nrf2* dimerization partners. The Neh2 domain contains two sites, named as *DLG* and *ETGE*, which combine with the cytoplasmic protein *Keap1*, a negative regulator of *Nrf2* transcriptional activity. Neh3–5 can bind with coactivating factors and are transactivating structural domains of *Nrf2*.Neh6 is a serine-rich region associated with the negative regulation of *Nrf2* stability, which relies on *Keap1*. Neh7 containing the retinoid X receptor inhibits the transcriptional activity of *Nrf2*

*Nrf2* is a member of the *Cap-n-Collar* (*CNC*) regulatory protein family and is a transcription factor with a highly conserved basic leucine zipper structure. *Nrf2* is a regulatory protein containing seven domains, Neh1–7, and has diverse features (Fig. 1). The Neh1 domain consists of the *CNC-bZIP* region, in which DNA binds to *sMaf* proteins as *Nrf2* dimerization partners [24, 25]. The Neh2 domain contains two sites, namely *DLG* and *ETGE*, which combine with the cytoplasmic protein *Keap1*, a negative regulator of *Nrf2* transcriptional activity [26]. Neh3–5 can bind to coactivating factors and are transactivating structural domains of *Nrf2 *[27, 28]. Neh6 is a serine-rich region associated with the negative regulation of *Nrf2* stability, which relies on *Keap1 *[29]. Neh7 containing the retinoid X receptor inhibits the transcriptional activity of *Nrf2 *[30].

**Fig. 2:**
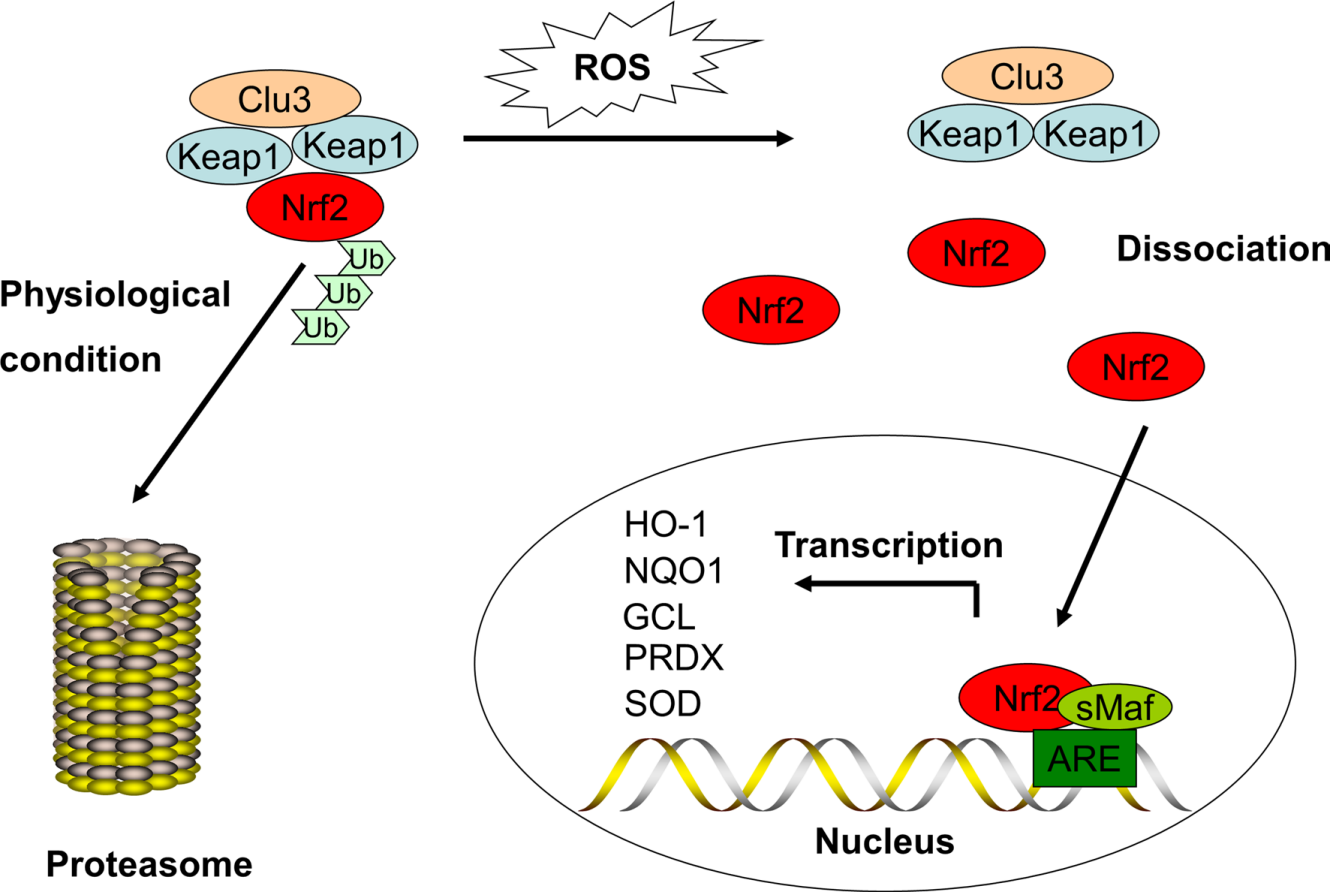
The classical view of *Nrf2* activation and response. Under physiological conditions, *Nrf2* is anchored in the cytoplasm by *Keap1* as a substrate for Cullin 3-dependent E3 ubiquitin ligase complex and can induce ubiquitination and promptly degrade *Nrf2* via the proteasome. However, when ROS or electrophiles attack cells, *Nrf2* detaches from *Keap1* and is rapidly transferred into the nucleus, forming a heterodimer with the *sMaf* protein and then integrating with the *ARE*, thereby transcriptionally activating *Nrf2*-regulated antioxidant gene expression including *HO-1,NQO1,GCL,PRDX* and *SOD* to exert anti-oxidative effects

Under physiological conditions, *Nrf2* is anchored in the cytoplasm by *Keap1* as a substrate for the cullin 3-dependent E3 ubiquitin ligase complex and can induce ubiquitination and promptly degrade *Nrf2* via the proteasome. However, when ROS or electrophiles attack cells, *Nrf2* detaches from *Keap1* and is rapidly translocated into the nucleus, forming a heterodimer with the *sMaf* protein and then integrating with the *ARE*, thereby transcriptionally activating *Nrf2*-regulated antioxidant gene expression including *HO-1,NQO1,GCL,PRDX and SOD* to exert anti-oxidative effects (Fig. 2). *Nrf2* has a short half-life of around 10–30 min, and thus the high turnover of *Nrf2* induced by *Keap1* maintains ultra-low levels of *Nrf2* [31, 32]. The protein products of these genes mediate detoxification through glutathione coupling and participate in ATP-dependent drug efflux, which may be involved with cisplatin resistance in OC [33]. High levels of *Nrf2* provide a protective environment in both normal and cancer cells.

Excessive activation of *Nrf2* is considered as an intermediate link in cell proliferation and causes drug resistance in cancer therapy as well [34–36]. To be specific, *Nrf2* activation and *Keap1* inactivation mutations are the precursors of permanent constitutive activation of the *Nrf2-*dependent AR pathway, which is frequently observed in cancer. Besides, anti-cancer radiation and chemotherapies, rely heavily on the production of ROS to induce cytotoxicity [37–39]. Hence, excessive activation of the *Nrf2*-dependent AR pathway will reduce the effectiveness of such treatments [40, 41].

A clinical study has indicated that high cytoplasmic *Nrf2* expression (the inactivated form of *Nrf2*) in serous carcinoma subtypes is associated with longer survival (p < 0.05), which appears to correlate with high ERα expression (p < 0.05) [42]. The same team found that *Nrf2* expression in the cytoplasm was positively correlated with PR expression (p < 0.05) [43]. Furthermore, a retrospective study of the relationship between *Nrf2* expression and clinical prognosis in 108 patients with different subtypes of OC showed that a high expression of *Nrf2* in OC indicates short DFS (HR: 2.084; 95% CI: 1.229–3.536) and OS (HR: 2.487; 95% CI: 1.443–4.286) [44]. Konstantinopoulos et al. found that among 64 advanced EOC patients, the upregulation of *Nrf2* promoted cisplatin resistance in OC patients and was associated with a short OS (P < 0.05) [45]. However, another study showed that chemoresistance is not significantly correlated with *Nrf2* expression, although patients with low *Nrf2* expression have higher recurrence rates and death rates than patients with high *Nrf2* expression. [46] Hence, further studies on the relationships between clinical prognosis and *Nrf2* expression, as well as relevant drug resistance mechanisms related to *Nrf2*, are needed.

## Effect of *Nrf2 *on treatments for OC

### Oncogenic mutations in OC may promote drug resistance by activating *Nrf2*

Disorder of *Nrf2/Keap1* caused by mutation and activation of up-stream oncogenes is associated with nuclear transportation and constitutive activation of *Nrf2*. Gina et al. have confirmed that oncogenic mutations in primary murine cells, such as *Kras, Braf and Myc*, separately increased the constitutive transcription of *Nrf2* to stabilize the basal *Nrf2* level and hence reduce intracellular ROS, ultimately causing cells to escape from apoptosis and promoting tumorigenesis, metastasis and chemoresistance [9, 47]. In view of the relationship of ROS and *Nrf2* with tumorigenesis, *Nrf2* appears to be a significant target for cancer treatment.

### Role of *Nrf2* in ROS-mediated therapy resistance in OC

#### Role of *Nrf2* in ROS-mediated cisplatin resistance in OC

As mentioned earlier, ROS play an indispensable role in the development of drug resistance. As the main antioxidant regulator, *Nrf2*, which is involved in ROS detoxification, tightly regulates drug resistance of tumors. It has been reported that during oxidative stress, as the transcription target of *Nrf2*, *p62/SQSTM1* competes with *Nrf2* for binding to *Keap1* and forms a positive feedback loop between *p62* and *Nrf2 *[48]. Xia et al. showed that overexpressed *p62* may protect cells from vitamin K3-induced ROS generation by up-regulating antioxidant genes downstream of *Nrf2*, including *HO-1* and *NQO1*, in OC [49]. Additionally, recent cases reported by Wu et al. also support the hypothesis that overexpression of CD99, a significant downstream gene of *Nrf2*, facilitates *Nrf2*-mediated cisplatin resistance in OC [50, 51]. Bao et al. suggested that low levels of *Nrf2* suppressed the expression of *ABCF2* and enhanced cisplatin sensitivity in OC cells by mediating the drug efflux pump mechanism [52]. Chen et al. argued that knockdown of *Nrf2* in the SKVO3 cell line increased the production of ROS induced by cisplatin by increasing the phosphorylation level of *p38-JNK*.This subsequently led to elevation of *ATF2* levels, followed by decreased expression of *AKR1C1*,which is involved in apoptosis, ultimately promoting the sensitivity of OC to cisplatin [53]. It was recently reported that activation of *Nrf2* promotes activation of its downstream gene *AKR1C1*, which converts progesterone to an inactive form and promotes platinum resistance in ovarian cancer, while metformin reverses this process by increasing PR expression [54]. Mechanistically, Sun et al. found that *SIRT5* contributes to the cisplatin resistance of OC by inhibiting cisplatin-mediated DNA damage via ROS through *Nrf2* pathway modulation [55].* SLC40A1*, as a novel iron metabolism-associated gene, serves as the only iron exporter gene with several putative *Nrf2* binding sites. Wu et al. found that *Nrf2* is highly expressed in cisplatin-resistant OC cells. Significantly increased gene expression of *SLC401*, a transferrin that inhibits *Nrf2* translocation into the nucleus, reverses iron overload—induced cisplatin resistance in OC cells [56].

#### Molecular factors involved in *Nrf2* regulation contribute to paclitaxel resistance

Paclitaxel is a first-line adjuvant drug for the treatment of OC, but only about half of OC patients respond to it [57, 58]. It is a new anti-microtubule drug that promotes tubulin polymerization to inhibit depolymerization, keeps tubulin stable, and inhibits cell mitosis. These different mechanisms cause a cascade of toxic effects in OC, such as the reduction of *Δψm* or elevation of ROS, which will eventually lead to cell death [59]. Enhancing the sensitivity of OC patients to paclitaxel is of great significance to improve prognosis. Stimulation of *NADPH* oxidase to accumulate ROS is an important part of paclitaxel cytotoxicity in cancer cells [60, 61]. Woo et al. held the view that inhibition of *Nrf2* can enhance the chemosensitivity of cancer cells to paclitaxel [62]. We have reason to believe that targeting *Nrf2* levels in OC cells may play an important role in overcoming paclitaxel resistance.

#### Role of *Nrf2* in ROS-mediated PARP inhibitor sensitivity in OC

At present, under the condition of platinum resistance in OC, PARPi have shown encouraging effects in the first [63–65] and second-line [66, 67] maintenance therapy for patients with *BRCA1/2* mutation and *HRD *[68]. Cells with HRD must depend on the replaceable mechanisms of *NHEJ* and *BER*, both of which require PARP enzymes [69]. *BRCA1/2* mutant cancer cells may develop PARPi resistance by restoring *HR* repair and/or protecting replication forks [70].

Mitochondrial metabolism and ROS production cause DNA oxidative damage and genomic instability in cancers [71]. *HRD* OCcells require high levels of NAD + and ATP to power PARP-dependent DNA repair [72]. Besides, some scholars have found that PARPi enhanced the effect of *Nrf2* inhibitors in *BRCA1-*mutant OC cells without fear of side effects from the combination of *Nrf2* inhibitors with chemotherapeutics [73]. From the above findings, we can speculate that *Nrf2* may play an irreplaceable role in PARPi repair of ROS-DNA oxidative damage.

#### Role of *Nrf2* in ROS-mediated pertuzumab and trastuzumab resistance in OC

Several studies have proved that *HER2/HER3*, *Nrf2*,and ROS play a key role in promoting growth and drug resistance in cancer cells [74–79].  Specifically, as a key regulator of the *Nrf2* pathway, ROS can regulate the *HER2/HER3* complex and activate its function. When pertuzumab and trastuzumab, which target *HER2/HER3 r*eceptors, are used to treat with OC cell lines, *Nrf2* inhibition suppress the *Nrf2*-dependent antioxidant response pathway, thereby allowing OC cells to overcome resistance to monoclonal antibodies. Khalil et al. proved that *Nrf2* is a key factor driving the drug resistance in OC; this provides a new treatment idea in the sensitization of OC to immune targeted therapy [80].

*Nrf2* inhibition increases the sensitivity of OC cells to adriamycin, one of the chemicals used in the treatment of OC [81]. Besides, *Nrf2* modulates the sensitivity of OC cells to lapatinib and erlotinib by regulating the *HER1* receptor [82].

#### Role of  *Nrf2* in ROS-mediated Mppa-PDT resistance in OC

PDT is a new type of tumor treatment method that has emerged in response to the development of medicine. It uses a photosensitizer that specifically accumulates in tumor tissues—currently, Mppa has a wide range of clinical application prospects due to its good absorption, high energy density, and strong permeability [83, 84]. It is activated under a specific wavelength of light, and a complex photochemical reaction occurs to generate ROS, which lead to irreversible tumor damage [85–87]. According to a previous research, *Nrf2* silencing enhanced PDT sensitivity in breast, colon, renal, and glioblastoma cancer cells based on Mppa, which can increase the accumulation of photosensitizers by down-regulating *ABCG2*, thereby promoting the production of ROS [88]. Coincidentally, Tian et al. found that the inhibition of *Nrf2- ABCG2 /HO-1* signaling increased ROS levels by attenuating antioxidants or pumping Mppa out of OC cells—suggesting that *Nrf2-ABCG2* signaling might be involved in the intrinsic resistance to Mppa-PDT [89].

#### Role of *Nrf2* in ROS-mediated ferroptosis resistance in OC

Ferroptosis is a novel mode of cell death first discovered by Dixon et al. in 2012 that is,—associated with unique morphological structure, biochemical, and genetic manifestations; it is essentially oxidative damage caused by excessive accumulation of iron ion-dependent lipid peroxidation products, mainly mitochondrial alterations [90]. Under normal conditions, *Nrf2* remains inactive; when induced by ROS stimulation or electrophile substances, *Nrf2* changes its molecular conformation and activates downstream antioxidant enzymes to play the role of an antioxidant and inhibit cellular ferroptosis [91]. There are two pathways to synthesize glutathione, which plays an essential role in combating oxidative stress, reducing lipid peroxidation, and protecting tissue cells, —in tumor cells: (a) The classical XC-system: the key factor is *SLC7A11*; and (b) Reverse transsulfuration pathway, and the key enzyme in this pathway is *CBS*; The above pathways can be activated by the ability of *GPx4* to specifically convert highly toxic lipid hydrogen peroxide to non-toxic lipid alcohols, breaking down hydrogen peroxide to water, and its inactivation can induce excessive production of lipid ROS, which can contribute to ferroptosis. It has been reported that *GPx4* is an *Nrf2* downstream gene and that *Nrf2* upregulation or *GPX4* overexpression may be significantly associated with ferroptosis resistance in head and neck cancer, but this has not been confirmed in OC [92, 93]. In addition, Liu et al. showed that in OC, *Nrf2* also causes erastin-induced ferroptosis resistance by activating *CBS *[94].

## Natural inhibitors of *Nrf2*

Given that *Nrf2* has a protective effect on tumors and can cause chemotherapy resistance, in recent years, many chemical substances and plant extracts have been reported to inhibit *Nrf2* to confront the problem of drug resistance [95–100].

Brusatol, a quassinoid compound derived from Brucea javanica, is considered as a general translation inhibitor that results in decreased levels of short-lived proteins including *Nrf2 *[95]. For this reason, brusatol’s ability to overcome chemoresistance is compromised. Recently, Chen et al. isolated a *plCSA*-binding peptide from the malaria protein *VAR2CSA*, which effectively promotes the binding of brustol to OC, thus overcoming the drawback mentioned above [96]. In addition, Cucci et al. showed that ailanthone from *Ailanthus altissima* could significantly inhibit the expression of *Nrf2* and *YAP* protein and subsequently inhibit the growth and colony formation of cisplatin-sensitive and cisplatin-resistant OC cells, and exert greater inhibitory effects on the migration of targeted cisplatin-resistant cells [97].

There are also some compounds that have not been proven in OC. Ascorbic acid, an inhibitor of *Nrf2*, partially restored cell sensitivity to imatinib by down-regulating *Nrf2* and reducing the expression of* γ-GCSL* and the level of glutathione [98], and increased the sensitivity of HeLa cells to cisplatin and adriamycin [99]. Apigenin, a flavonoid extracted from various vegetables and fruits, inhibits the *Nrf2* pathway, thereby making doxorubicin-resistant liver cancer cells sensitive to doxorubicin and increasing intracellular doxorubicin [100].

## Conclusions

The *Keap1-Nrf2-ARE* system is a critical defense mechanism to protect cells from oxidative stress and electrophilic stress. While temporary *Nrf2* activation during stress is advantageous for cell proliferation [101], sustained *Nrf2* activation in cancer cells confers chemoresistance and aggressive tumorigenic activity, which has deleterious effects on the cancer patients [102–105]. Since *Nrf2* increases the antioxidant and detoxification capacity of cancer cells, sustained high levels of *Nrf2* activity can enhance therapeutic resistance of cancer cells. *Nrf2* also drives metabolic reprogramming and cooperates with other oncogenic pathways to establish cellular metabolic processes that favor cell proliferation.

**Fig. 3:**
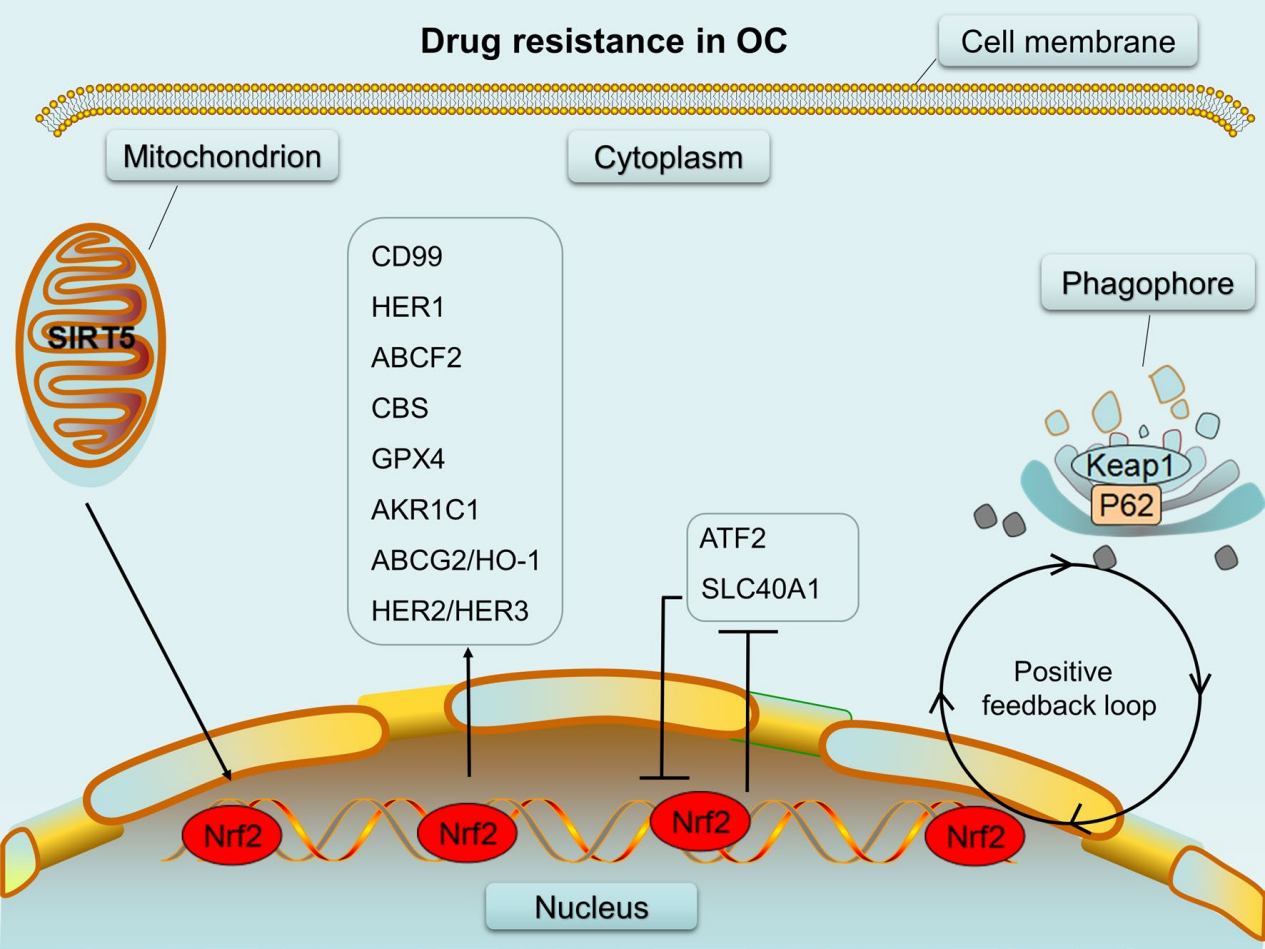
Various drug resistance mechanisms associated with *Nrf2*. *SIRT5,CD99,ABCG2/HO-2,HER1,HER2/HER3,ABCF2,GPX4,AKR1C1* and *CBS* have a positive relationship with *Nrf2* as molecules regulated by *Nrf2* or regulating *Nrf2*;while ATF and SLC40A1 have a negative relationship with *Nrf2*; As the transcription target of *Nrf2*, *p62/SQSTM1* competes with *Nrf2* for binding with *Keap1* and form a positive feedback loop between *p62* and *Nrf2*.(“→” represents “activation” “— ” means “inhibition”.)

Most patients with OC treated by chemotherapy, immunotherapy, and molecular targeted therapy eventually develop resistance and show poor outcomes. In fact, there are many proteins that regulate the process of drug resistance in OC;—for example, downregulation of 14-3-3ζ, a key protein involved in ovarian development and gamete function [106–108], by RNA interference in OC cells results in enhanced sensitivity to cisplatin-induced cell death [109]. Meanwhile, multiple isoforms of 14-3-3 protein strongly interact with the cell cycle protein *CDC25B*, which is inactivated in *Nrf2*^−^/^−^ cells, to regulate cell cycle in oocyte [110, 111]. Why did we choose to review *Nrf2* as a key pivot in the regulation of drug resistance in OC? As described above, *Nrf2*, as the main regulator of the antioxidant response pathway, has received increasing attention for its significant effect in drug-resistant OC and thus, may be targeted for treating advanced OC. So far, several *Nrf2* inhibitors have been used for overcoming drug resistance in OC. In addition to *Nrf2* inhibitors, new potential therapeutic targets related to *Nrf2* for overcoming drug resistance in OC are being identified (Fig. 3; Table 1). However, the mechanisms of *Nrf2*-associated drug resistance in OC cells remain unclear and should therefore be further investigated. There is also a need to develop appropriate animal models to evaluate the therapeutic efficacy of *Nrf2*-related therapeutic targets in drug-resistant OC.

**Table 1:** Overview of *Nrf2*-interacting factors in cancer cell lines

Gene	Effects on *Nrf2*	Effects on cell	Tumor model	Resistance to	References
P62	Activator	Protective	SKOV3/DDP	Cisplatin	[48]
CD99	Activator	Protective	A2780,COC1/DDP	Cisplatin	[50, 51]
ABCF2	Activator	Protective	A2780	Cisplatin	[52]
ATF2	Inhibitor	Cytotoxic	SKOV3	Cisplatin	[53]
AKR1C1	Activator	Protective	–	Platinum	[54]
SIRT5	Activator	Protective	A2780,SKOV3,CAOV3	Cisplatin	[55]
SLC40A1	Inhibitor	Cytotoxic	A2780CP,PEO4,COC1/DDP	Cisplatin	[56]
HER1	Activator	Protective	PEO1, SKOV3, and OVCAR3	lapatinib and erlotinib	[82]
HER2/HER3	Activator	Protective	PEO4,OVCAR4,SKOV3	Pertuzumab/Trastuzumab/Docetaxel	[80]
ABCG2	Activator	Protective	SKOV3	Mppa-PDT	[89]
GPX4	Activator	Protective	AMC-HN2–11/SNU	Ferroptosis	[92, 93]
CBS	Activator	Protective	SKOV3 and OVCA429	Ferroptosis	[94]
Compounds					
Brusatol	Inhibitor	Cytotoxic	SKOV3/HEC-1-A/A549	–	[96]
Ailanthone	Inhibitor	Cytotoxic	A2780/CP70	Cisplatin	[97]
Ascorbic acid	Inhibitor	Cytotoxic	KCL22/SR; Hela	Imatinib;cisplatin / adriamycin	[98, 99]
Apigenin	Inhibitor	Cytotoxic	BEL-7402/ADM	Doxorubicin	[100]

Besides active exploration and mechanistic research on therapeutic targets associated with *Nrf2*, studies for discovering diagnostic biomarkers and surrogate markers for refractory OC are also needed. For progress in diagnosis and treatment, further researches and technical improvements are required. Consequently, a thorough elucidation of the function of *Nrf2 *will help to improve the clinical diagnosis and prognosis of OC.

## Data Availability

Not applicable.

## Abbreviations

OC: Ovarian cancer
Nrf2: Nuclear erythroid-related factor-2
Keap-1: Kelch-like ECH-associated protein-1
sMaf: Small musculoaponeurotic fibrosarcoma
ROS: Reactive oxygen species
bZIP: Basic leucine zipper
HO-1: Heme oxygenase 1
NQO1: NAD(P)H dehydrogenase (quinone) 1
ABCG2: ATP-binding cassette, subfamily G, member 2
AKR1C1: Aldo-keto reductase family 1 member C1
SIRT5: Sirtuin 5
SLC40A1: Solute carrier family 40 member 1
TME: Tumor microenvironment
Mppa-PDT: Methyl pyropheophorbide-amediated photodynamic therapy
ABC: ATP-binding cassette
P-gp: P-glycoprotein
PARPi: Poly-ADP Ribose Polymerase inhibitors
HRD: Homologous recombination deficiency
CBS: Cystathionine β-synthase
